# Genetic determinants of antimicrobial resistance in three multi-drug resistant strains of *Cutibacterium acnes* isolated from patients with acne: a predictive *in silico* study

**DOI:** 10.1099/acmi.0.000404

**Published:** 2022-08-11

**Authors:** Catriona Beirne, Emily McCann, Andrew McDowell, Georgios Miliotis

**Affiliations:** ^1^​ Antimicrobial Resistance and Microbial Ecology Group, School of Medicine, National University of Ireland, Galway, Ireland; ^2^​ Nutrition Innovation Centre for Food and Health, (NICHE), School of Biomedical Sciences, Ulster University, Coleraine, Ireland

**Keywords:** antimicrobial resistance, *Cutibacterium acnes*, gyrA, pYU39, YfmO efflux

## Abstract

**Objectives.:**

Using available whole genome data, the objective of this *in silico* study was to identify genetic mechanisms that could explain the antimicrobial resistance profile of three multi-drug resistant (MDR) strains (CA17, CA51, CA39) of the skin bacterium *Cutibacterium acnes* previously recovered from patients with acne. In particular, we were interested in detecting novel genetic determinants associated with resistance to fluoroquinolone and macrolide antibiotics that could then be confirmed experimentally.

**Methods.:**

A range of open source bioinformatics tools were used to ‘mine’ genetic determinants of antimicrobial resistance and plasmid borne contigs, and to characterise the phylogenetic diversity of the MDR strains.

**Results.:**

As probable mechanisms of resistance to fluoroquinolones, we identified a previously described resistance associated allelic variant of the *gyrA* gene with a ‘deleterious' S101L mutation in type IA_1_ strains CA51 (ST1) and CA39 (ST1), as well as a novel E761R ‘deleterious’ mutation in the type II strain CA17 (ST153). A distinct genomic sequence of the efflux protein YfmO which is potentially associated with resistance to MLSB antibiotics was also present in CA17; homologues in CA51, CA39, and other strains of *Cutibacterium acnes *, were also found but differed in amino acid content. Strikingly, in CA17 we also identified a circular 2.7 kb non-conjugative plasmid (designated pCA17) that closely resembled a 4.8 kb plasmid (pYU39) from the MDR *Salmonella enterica* strain YU39.

**Conclusions.:**

This study has provided a detailed explanation of potential genetic determinants for MDR in the *Cutibacterium acnes* strains CA17, CA39 and CA51. Further laboratory investigations will be required to validate these *in silico* results, especially in relation to pCA17.

## Impact statement

*Cutibacterium acnes* is an opportunistic pathogen known for its association with the inflammatory skin condition acne vulgaris. More recently, it has also been recognised for its potential role in medical device-related infections and other clinical conditions. The widespread use of oral and topical antibiotics to treat acne has led to the widespread emergence of multidrug resistant (MDR) forms of *Cutibacterium acnes *. Although resistance to tetracyclines and MLSB (Macrolide-Lincosamide-Steptogramin-B) antibiotics frequently occurs via single nucleotide polymorphisms (SNPs) in rRNA, the role of other potential genetic mechanisms has received less attention. In this study, we performed a complete *in silico*-based genomic characterisation of three strains of *Cutibacterium acnes* (CA17, CA39 and CA51), isolated from acne patients in China; these strains were resistant to clindamycin, erythromycin and moxifloxacin. While highlighting a number of previously described genetic elements known to be associated with MLSB and fluoroquinolone resistance, we also describe a novel SNP that may contribute to fluoroquinolone resistance and propose yet unidentified multidrug efflux pumps as potential resistance determinants. We also genetically showcase a rare plasmid transfer between MDR *Salmonella enterica* and an MDR *Cutibacterium acnes* strain.

## Data Summary

All data used in this study are publically available from the National Centre for Biotechnology Information (NCBI). The assemblies for the three MDR *Cutibacterium acnes* strains CA-17; CA-51 and CA-39 are publically available on NCBI under Biosamples SAMN07259472, SAMN07268412 and SAMN07268976 respectively. A complete listing of all strains, plasmids and key proteins used in this study is contained in (Table S1, available in the online version of this article).

## Introduction

*Cutibacterium acnes* is a Gram-positive anaerobic bacterium that predominately colonises regions of the skin with high concentrations of sebaceous glands, namely the back, scalp and face [1]. It can, however, also be isolated from other sites including the oral cavity, gastrointestinal and genitourinary tracts and prostate tissue [2]. As part of the normal human microbiota, *Cutibacterium acnes* and other cutibacteria help to maintain skin homeostasis via the release of short chain fatty acids and other biological molecules that inhibit the growth and colonization of the skin by more pathogenic species, such as *Staphylococcus aureus* [3].

Despite the important role *Cutibacterium acnes* plays in skin health, it can also behave as an opportunistic pathogen linked to a number of diseases occurring on and beyond the skin [4]. Most notably, it has a long association with the very common skin condition acne vulgaris which occurs during adrenarche and results in the development of papules, pustules or nodules during its inflammatory phase [5, 6]. Our understanding of the underlying pathophysiology of acne vulgaris has greatly improved over the past 15 years mostly due to our improved characterisation of the population genetic structure of *Cutibacterium acnes* [7]. Rather than simple *Cutibacterium acnes* overgrowth, the now emerging view is that loss of *Cutibacterium acnes* phylotype diversity on the skin is responsible, in-part, for the development of acne. In particular, a dysbiotic shift that results in a pre-dominance of strains from the type IA_1_ phylogroup of *Cutibacterium acnes* subsp. *acnes* appears to play a primary role in acne pathogenesis [8].

For decades, the mainstay of treatment for moderate-to-severe acne has been the use of oral and topical antibiotics, including, macrolides, tetracyclines, and lincosamides [9, 10]. The prolonged overuse of antibiotics in acne treatment has, however, led to an increase in circulating antibiotic resistant *Cutibacterium acnes* strains globally, even amongst individuals with no history of acne [11]. As a result, it is probable that in the future more patients will develop recalcitrant acne in primary care due to antibiotic resistance, thus requiring referral to specialist dermatology clinics for treatment. Furthermore, the increasing emergence of antibiotic-resistant strains of *Cutibacterium acnes* may also impact on other non-skin-related diseases where this bacterium is implicated.

Antibiotic resistance in *Cutibacterium acnes* is primarily driven by chromosomal mutations in rRNA genes, with single nucleotide polymorphisms (SNPs) in the 16S rRNA gene responsible for tetracycline resistance, while SNPs in the 23S rRNA gene are a common cause of MLSB (Macrolide-Lincosamide-Steptogramin-B) resistance phenotypes [12–15]. Less commonly, resistance can also occur via horizontal gene transfer (HGT) of antimicrobial resistance genes (ARGs) present in plasmids, integrons or transposons. Acquisition of the transposon Tn5432 which carries the *erm(X*) gene has also been reported as another cause of MLSB resistance in *Cutibacterium acnes* [15, 16], as well as a multidrug resistant plasmid pTZC1 carrying the macrolide-clindamycin resistance gene *erm50* [17]. Although quinolones are not a first line drug for anaerobic infections, they are used for polymicrobial infections, such as bone and joint infections where *Cutibacterium acnes* pathogenicity has been implicated [18]. While fluoroquinolone resistance in *Cutibacterium acnes* has not been extensively explored at the molecular level, amino acid substitutions in the quinolone resistance-determining regions (QRDR) of the gyrase (*gyrA*) and topoisomerase IV (*parC*) genes seem to be the underlying cause [19, 20].

The recent widespread use of whole genome sequencing has greatly enhanced molecular surveillance of antibiotic resistance and associated molecular mechanisms. Despite this, the number of whole genome analyses of antibiotic-resistant strains of *Cutibacterium acnes* have been small and limited to published announcements rather than detailed genetic analyses. In 2017, a genome note by Zhang *et al*. [21] described the whole genome sequence (WGS) results for MDR strains of *Cutibacterium acnes* (CA17, CA39 and CA51) isolated from acne patients in Shanghai, China. These three strains were found to be resistant to clindamycin (MIC >128 mg l^−1^) erythromycin (MIC >128 mg l^−1^) and moxifloxacin (MIC=2 mg l^−1^). While the presence of the *erm(X*) gene in CA39 and CA51 likely explains the MLSB resistance phenotype in these strains, which had wild-type rRNA gene sequences, the molecular basis of fluoroquinolone resistance was not evident. Furthermore, no explanation for the mechanisms driving resistance in the CA17 strain were identified, warranting further study of this strain as previously recommended [21].

As a consequence, in this study our primary aim was to conduct a complete *in silico* genomic analysis of these three *Cutibacterium acnes* strains, with a focus on CA17, to better understand the genetic basis of their MDR, and identify potentially novel resistance mechanisms that can develop in *Cutibacterium acnes* due to antibiotic pressures.

## Methods

### Bacterial strains

In addition to CA17, CA39 and CA51, the genomes of a further seven *Cutibacterium acnes* strains available at the European Nucleotide Archive (ENA) /National Centre for Biotechnology Information (NCBI) were included in our study for comparative purposes. These strains were selected to cover the different phylogroups of the bacterium and clinical associations (Table 1). Apart from the type IC strain Prp38, these strains are not known to be antibiotic resistant for tetracycline or MLSB antibiotics; Prp38 has resistance due to known point mutations in 16S and 23S rRNA genes [22].

**Table 1.** Characteristics of the *Cutibacterium acnes* strains analysed in this study

Strain	Subspecies	Phylogroup	Ribotype	ST (CC)	SLST	Source	Biosample ID
CA17	*defendens*	Type II	2	153 (CC72)	K1	acne	SAMN07259472
CA39	*acnes*	Type IA_1_	1	1 (CC1)	A2	acne	SAMN07268976
CA51	*acnes*	Type IA_1_	1	1 (CC1)	A2	acne	SAMN07268412
HL025PA1	*acnes*	Type IA_1_	1	4 (CC4)	D1	healthy skin	SAMN00189202
P.acn17	*acnes*	Type IA_2_	3	22 (S)	F5	subcutaneous abscess	SAMN02602999
KPA171202	*acnes*	Type IB	1	5 (CC5)	H2	plate contamination	SAMN08348521
PRP38	*acnes*	Type IC	5	70 (CC107)	G1	acne	SAMN02469319
ATCC11828	*defendens*	Type II	2	27 (S)	K9	subcutaneous abscess	SAMN02602997
HL110PA3	*defendens*	Type II	6	7 (CC6)	K2	healthy skin	SAMN00189249
HL110PA4	*defendens*	Type II	6	7 (CC6)	K2	healthy skin	SAMN00189250

S=singleton.

### *In silico* typing

All strains were typed using the previously described *Cutibacterium acnes* Multilocus Sequence Typing (MLST_8_) and Single Locus Sequence Typing (SLST) schemes [22, 23]. MLST_8_ sequence types (STs) and related clonal complexes (CCs) were assigned at PubMLST (https://pubmlst.org/), while STs for SLST were determined at http://medbac.dk/slst/pacnes. Ribotypes (RT) were determined by Basic Local Alignment Search Tool (blast) analysis of 16S rDNA sequences against known *Cutibacterium acnes* RT sequences available at NCBI. Multiple sequence alignments were performed in Linux using muscle v 3.8.31–4 [24], and bootstrapped maximum likelihood phylogenetic trees inferred with RAxML v 8.2.1 [25]. The generated trees were visualized and edited using the Interactive Tree of Life (iTOL) v 5.0 tool [26].

### Core and accessory genome

Genome assemblies in FASTA format for the ten strains were annotated using PROKKA v 1.14.5 [27]. The GFF3 format annotated assemblies created by PROKKA were used as input for the generation of a core and accessory genome using the Linux native tool Roary v 3.13 [28]. Roary was used with a minimum 95 % identity for BLASTp. A gene presence absence matrix was created using the Python script roary_plots.py (v 0.1.0) [29]. A newick tree was generated based on core genome alignment using FastTree v2.1.1.0. As before, the generated tree was visualized and edited using (iTOL) v 5.0 [26].

### Genetic determinants of resistance

Resistance Gene Identifier (RGI) (Linux version 5.2.0) was used to predict antibiotic resistance genes (ARGs) in the three CA strains (CA51, CA39, CA17) [30]. Given the absence of any obvious genetic determinants, RGI analysis was performed using the ‘Loose’ (a.k.a. discovery) parameter and reference data from the Comprehensive Antibiotic Resistance Database (CARD) v 3.1.2. The RGI tool uses CARD’s protein homolog models (DIAMOND bitscore cut-offs) to detect functional homologs of AMR genes [31]. The results were filtered with >50 % identity, and >90 % sequence length used as cut-offs. Pairwise alignment between the multidrug efflux protein YfmO identified in CA17 (GenBank accession no. PIS93044.1) and that of *B. subtilis* (GenBank accession no. O06473.1) was performed using the EMBOLL-matcher [32]. Pairwise alignment between the HypS repressor of *Mycobacterium smegmatis* (GenBank accession no. ABK72338.1) and the MerR family transcriptional repressor homologue in CA17 (GenBank accession no. PIS94143.1) was also performed using EMBOSS-Needle. Both alignments were visualised using BoxShade v 3.21.

### Variant calling and protein effect evaluation

The Linux version of Snippy v 4.6.0 was used to identify SNPs with default parameters [33]. The results were manually curated and the SNP’s causing missense mutations in proteins known to result in MLSB or quinolone resistance phenotypes isolated. The curated missense mutations were used as input for PROVEAN in order to determine their predicted effect on the protein [34, 35]. PROVEAN was also run with default parameters, and with a cut-off score for a deleterious mutation set at −2.5. A score below −2.5 was considered ‘deleterious’ for the protein’s function suggesting a strong effect.

### Analysis of plasmid borne contigs

In order to investigate the ‘plasmidome’ of all CA strains, the Linux tool Platon v.1.6 was used with the FASTA sequences of all ten genomes [36]. A plasmid map of pCA17 was created with the GCView tool v 1.36 [37], and the plasmid annotated using PROKKA [27]. Following identification of pCA17 (GenBank accession no.: NJFY01000001.1), a BLASTn analysis was performed to verify its presence in any other strains within the NCBI database. A predicted ‘hypothetical’ protein (279 amino acids; GenBank accession no. PIS94559.1) within pCA17 was characterised for its features, localisation and function using the MPI Bioinformatics tool kit [38] and InterProScan v 5.51–85.0 [39]. The protein was also annotated using SnapGene Viewer software [40]. Topological analysis was conducted with TMSEG [41] and PredictProtein [42]. Protein sequence searching was performed using HHblits (by HMM-HMM alignment) [43]. Motif scanning for the hypothetical protein was carried out using https://www.expasy.org.

### CRISPR-Cas system identification

The draft assembled genomes of the ten strains, in FASTA format, were used as input for the identification of CRISPR arrays and the Cas protein. The Linux native tool CRISPRCasFinder was used with default parameters [44, 45].

## Results

### *In silico* typing

A summary of the typing results for all three CA strains is shown in Table 1. Strains CA39 and CA51 belonged to the *Cutibacterium acnes* subsp. *acnes* type IA_1_ phylogroup with MLST_8_ genotype ST1 (CC1) (Fig. S1). CA17 was identified as a *Cutibacterium acnes* subsp. *defendens* (type II) strain with MLST_8_ genotype ST153 (17-4-2-4-4-6-10-46) (CC72) (Fig. S1). Consistent with these results, ribotyping and SLST typing also identified these strains within the type IA_1_ and type II groups (Table 1).

### Core and accessory genes

**Fig. 1.**
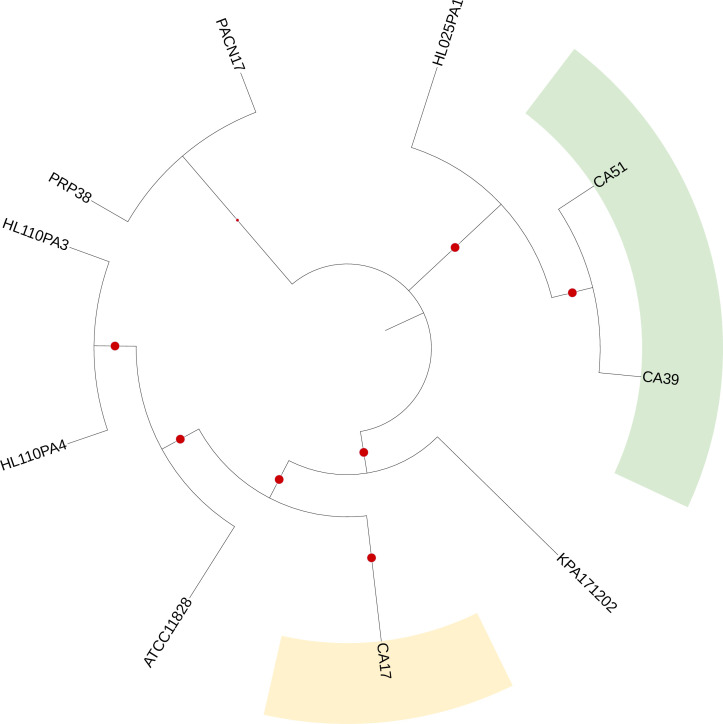
*
Cutibacterium acnes* cladogram illustrating the clustering of MDR strains CA17, CA39 and CA51 versus phylotype reference strains based on core genome phylogeny. Red circles represent bootstrap values ≥70 %.

Roary was used to provide an overview of the core and accessory genes of the three MRC strains versus seven other *Cutibacterium acnes* strains used for reference. Amongst the ten strains studied, a total of 3295 genes were identified and taken into consideration for this analysis. Consistent with MLST_8_ analysis, a cladogram based on core genome phylogeny(Fig. 1) clustered CA17 with *Cutibacterium acnes* subsp. *defendens* (type II) reference strains, while CA39 and CA51 clustered with *Cutibacterium acnes* subsp. *acnes* comparator organisms (type I).

**Fig. 2.**
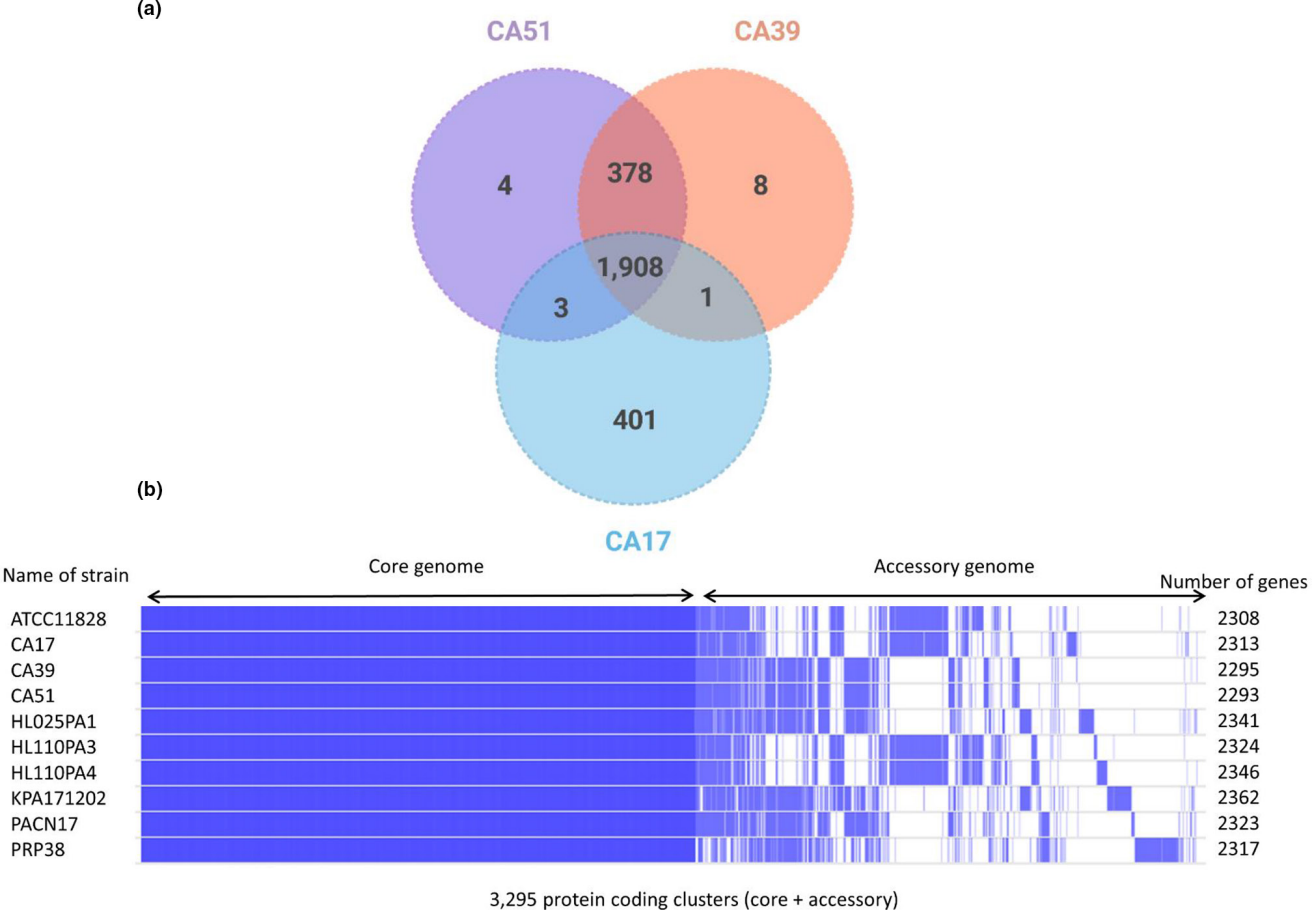
Venn diagram of shared or unique genes between MDR CA17, CA39 and CA51 strains based on pan genome analysis (a) schematic representation (Roary matrix) of genes present (blue) and (b) absent (blank) between CA and phylotype reference strains.

When the three MDR strains were compared based on their genetic content, the strain CA17 was found to have a higher number of genes (*n*=2313) versus CA39 (*n*=2293) and CA51 (*n*=2251), with a total of 1908 genes shared across all three strains (Fig. 2a).

With all ten strains, a total of 1728 genes formed the core genome with 1133 genes in the shell genome (present in 15–95 % strains). Lastly, 434 genes formed the cloud genome (<15 % strains) (Fig. 2b).

### Genetic determinants of resistance

To characterise the genome of CA17, we utilised RGI, SNIPPY and PROVEAN tools to investigate mechanisms underlying clindamycin, erythromycin and moxifloxacin resistance. We also analysed CA39 and CA51 for genetic elements that could explain fluoroquinolone resistance given that the previous description of *erm(X*) in these strains likely explained their MLSB resistance profile [21].

For the CA17 strain, RGI returned 130 predicted ARGs, with identities ranging from 19.9–73.5 %. A total of 12 ARGs passed the inclusion filter of >50 % identity and >90 % sequence length for further analysis. From these, three were predicted ARGs related to either fluoroquinolone (*gyrA* allelic variants) or MLSB (efflux pumps) resistance. In the former case, we examined the DNA gyrase and type IV topoisomerase subunits A and B, encoded by the *gyrA*, *gyrB*, *parC* and *parE* genes, respectively. The *gyrB*, *parC* and *parE* genes in CA17, CA39 and CA51 isolates did not possess any missense mutations when compared to their respective reference strains of the same subsp. However, the *gyrA* subunit in CA17 did display a unique amino acid composition versus all type II control strains (ATCC11828, HL110PA3 and HL110PA4) with three missense mutations, all of which were found to fall outside the QRDR region (positions 67–106) (Table 2). These mutations were only found in one other *Cutibacterium acnes* subsp. *defendens* strain, known as HMSC069G10, based on BLASTn analysis of all available closed and draft genomes deposited in NCBI; this strain was isolated from the eye of a patient and had the MLST_8_ genotype ST136 (17-4-15-4-4-6-10-12) (CC72). All other genomes were found to differ from CA17 and HMSC069G10 in the *gyrA* gene sequence. These missense mutations were subsequently used as input for PROVEAN to assess the effect they might have on *gyrA* function. Although two of the mutations were predicted to have an overall neutral effect, one SNP (G761R) was predicted to be strongly deleterious (PROVEAN score=−6.026) (Table 2).

**Table 2.** Missense mutations in genes related to quinolone and MLSB resistance relative to control strains

Strain(s)	Gene	Resistance	Amino acid substitution	PROVEAN score	Predicted effect	QRDR region
CA-17	*gyrA*	Quinolones	A670E	4.865	Neutral	−
			E761R	−6.026	Deleterious	−
			Q858K	−0.714	Neutral	−
CA-39; CA-51	*gryA*	Quinolones	S101L	−4.612	Deleterious	+
			M566V	2.942	Neutral	−
CA-17	YfmO	MLSB	A170T	−2.590	Deleterious	na
			G364S	2.590	Neutral	na

With strains CA39 and CA51, a previously described *gyrA* genotype associated with fluoroquinolone resistance in *Cutibacterium acnes* (S101L) was identified (within the QRDR region) when compared with the type IA_1_ reference strain HL025PA1 [20]; this genotype was classified as deleterious by PROVEAN analysis (Table 2). When compared with KPA171202 (type IB), a second missense mutation (M556V) was also detected (outside the QRDR), but this was classified as ‘neutral’ by PROVEAN (2.942) (Table 2).

**Fig. 3.**
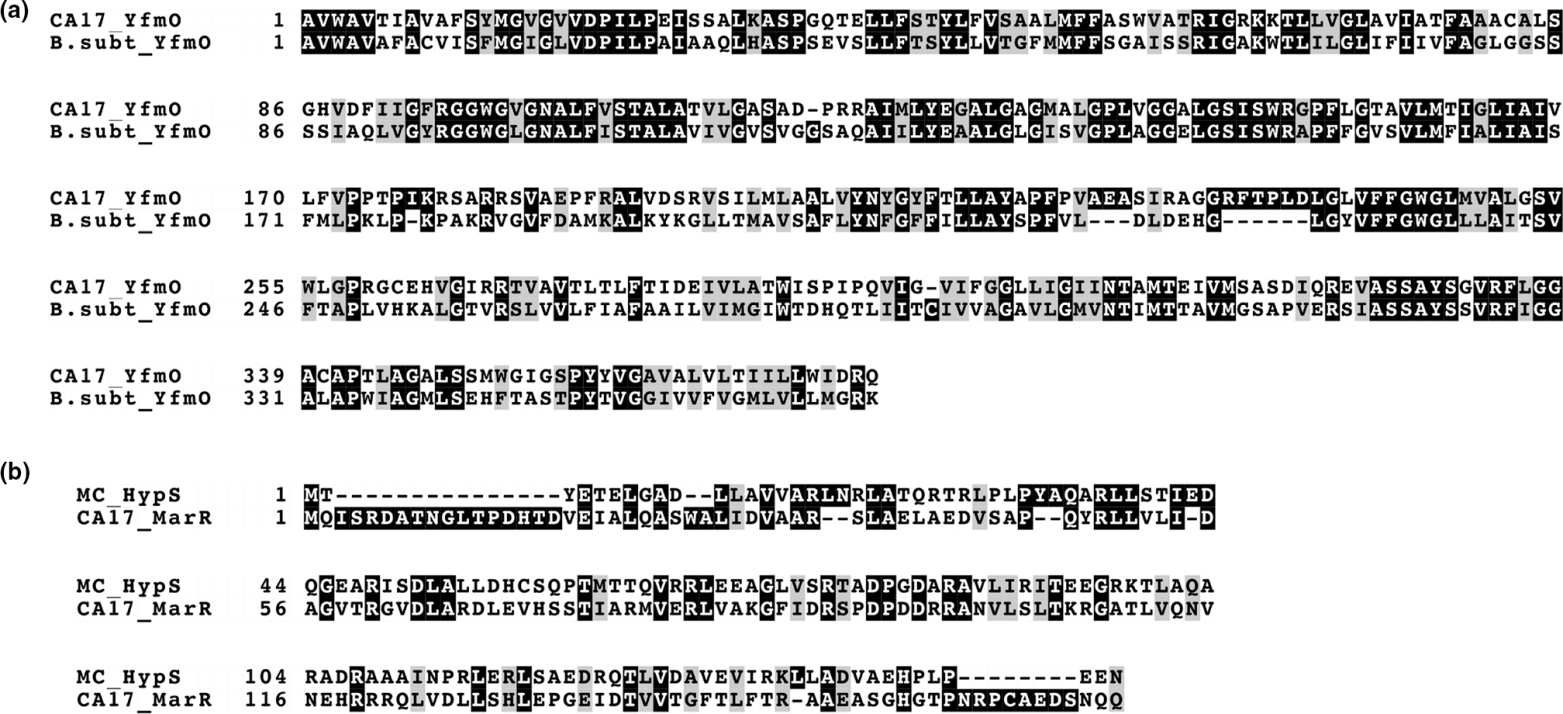
Pairwise alignments of the (a) YfmO protein identified in strain CA17 versus YfmO protein from MDR *B. subtilis *. **(b**) HypS repressor of *M. smegmatis* and the MerR family transcriptional repressor homologue in CA17.

**Fig. 4.**
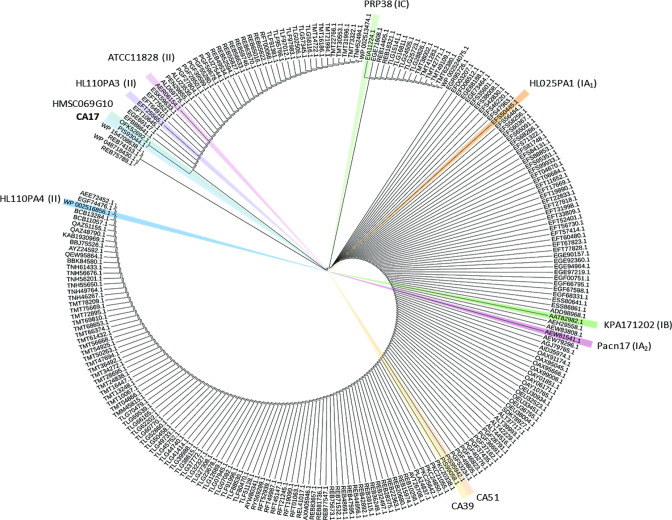
Phylogenetic tree of YfmO proteins in *Cutibacterium acnes *. The ten strains used in this study are highlighted. Note: sequences are from all whole genomes available in NCBI at the time of analysis.

While *erm(X*) was previously highlighted as the probable cause of MLSB resistance in CA39 and CA51, explanations for similar high levels of resistance in CA17 are lacking. Upon inspection of the CA17 genome, we identified the multidrug efflux protein YfmO (398 amino acids) (PIS93044.1), which is part of the major facilitator superfamily (MFS) and potentially associated with MLSB resistance. On the basis of pairwise alignment, this protein has a 43 % amino acid sequence identity and 64 % similarity to the multidrug-efflux protein YfmO originally identified in *B. subtilis* (Fig. 3a). While BLASTp analysis also revealed gene homologues of this efflux protein in most other *Cutibacterium acnes* strains deposited in NCBI, including CA51 and CA39 (which had identical sequences), only HMSC069G10 was found to have an identical amino acid composition to the sequence in CA17 (Fig. 4). The YfmO protein sequence was found to cluster into a small number of major clades across all whole genome sequences available in NCBI (Fig. 4). Two missense mutations (A170T, G364S) were the least number of *yfmO* gene differences identified between CA17 and other strains, including ATCC11828 and HL110PA3 type II control strains, and when we analysed these with PROVEAN they had scores of −2.590 (deleterious) and 2.590 (neutral) respectively. With the type II control strain HL110PA4, a greater number of amino acid differences were observed (96 % sequence identity; 381/397), including A170T and G364S. Compared to CA39 and CA51, the CA17 strain had 95.73 % sequence identity in the YfmO protein, with a total of 17 amino acid differences. Of these, only one (V84A) had deleterious scores on PROVEAN analysis (−3.641).

We also observed protein homologies of the YfmO protein in other cutibacteria versus CA17, with *Cutibacterium avidum* demonstrating very high identity (99 % coverage; 88–99 % identities), while *Cutibacterium namentense* (41 % coverage; 30 % identity), *Cutibacterium modestum* (41 % coverage; 31 % identity) and *Cutibacterium granulosum* (39 % coverage; 26 % identity) had coverage and identities that were much lower.

Given that the *Mycobacterium smegmatis* homologue of the YfmO MDR efflux functions as a redox-sensitive operon by utilising a MerR family transcriptional repressor (HypS), we also searched the CA17 genome for MerR family transcriptional repressor homologues. We identified one protein (GenBank accession no: PIS94143.1) that demonstrated 30.1 % identity to the HypS repressor of *Mycobacterium smegmatis* (GenBank accession no. ABK72338.1) (Fig. 3b).

### Identification of a plasmid borne contig originating from Enterobacteriaceae

**Fig. 5.**
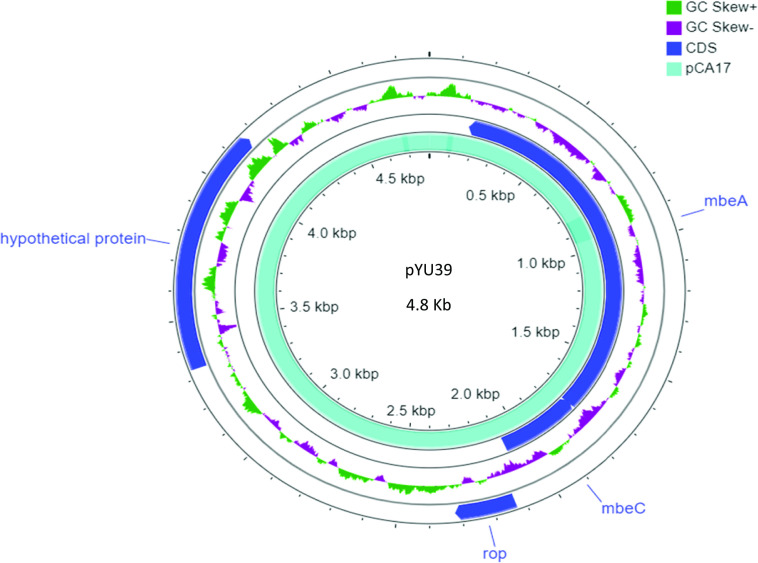
Comparison of pCA17 and pYU39 plasmid maps, including gene annotations.

**Fig. 6.**
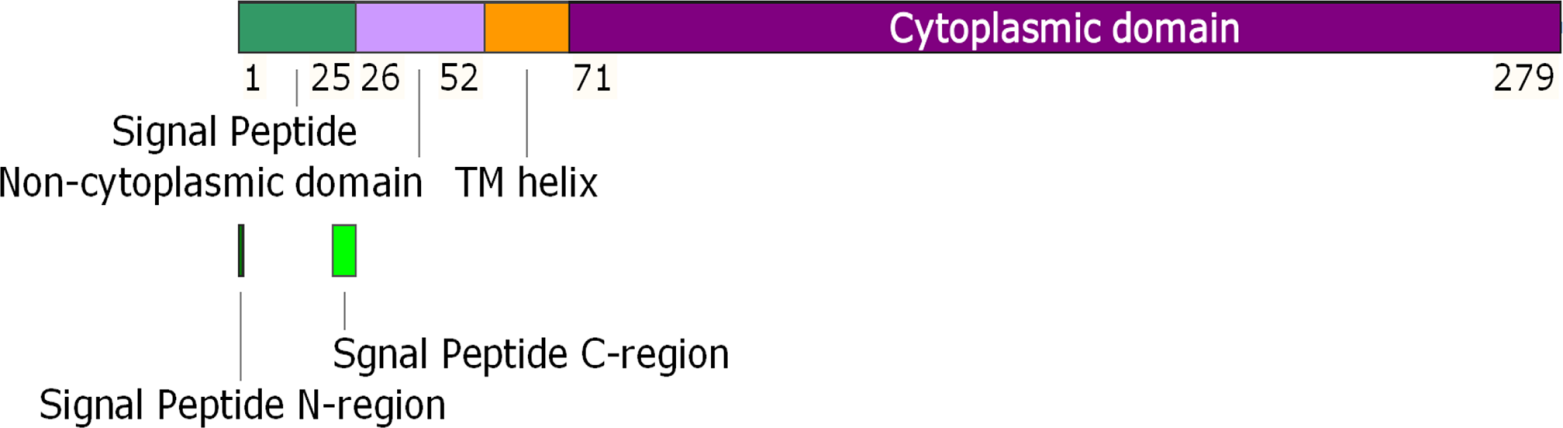
Illustration of domains identified on the putative transmembrane protein (279 amino acids long) present in pCA17.

Prediction analysis of plasmid borne contigs with Platon identified a single, circular plasmid of 4715 bp for strain CA17 (designated pCA17). pCA17 closely matched (99.9 % sequence identity; 98.2 % length coverage) a previously described 4.8 kb plasmid pYU39 from the MDR resistant, human invasive, *Salmonella enterica* strain YU39 (Fig. 5). pCA17 covered an entire contig (number 25) in the draft genome assembly of CA17 (GenBank accession no. NJFY01000001.1). BLASTn analysis revealed that this plasmid was conserved across Gram-negative Enterobacteriaceae with 31 highly similar plasmids (>98 % sequence identity, >98 % length coverage) previously identified in other clinical strains of *Salmonella enterica *, *Escherichia coli* and *Klebsiella pneuomoniae*. The plasmid was annotated and found to carry five protein-encoding regions and one non-coding RNA gene. These proteins included a regulatory protein (Rop), mobilisation proteins (MbeB, C and D) and a hypothetical protein with 99 % amino acid identity (100 % coverage) to homologues in *Salmonella enterica* and *Escherichia coli *, and 96 % identity with *Klebsiella pneumonia* (96 % coverage) based on BLASTp analysis (Fig. 5). In order to characterise this plasmid further, its predicted hypothetical protein (279 amino acids) was investigated for structure using HHblits (utilizing HMM-HMM alignments). This revealed that the protein contains transmembrane segments, a signal peptide and a coiled-coil segment; topological analyses also predicted the presence of a cytoplasmic domain, a transmembrane helix, a non-cytoplasmic domain and a signal peptide, further affirming its transmembrane nature (Fig. 6). Also, analysis of motifs generated strong matches to a prokaryotic membrane lipoprotein lipid attachment site (positions 1–15) and an EF-hand Ca^2+^-binding domain (position 167–179; DLSSEKYLSYKEI). From a molecular function perspective, the protein is predicted to act as a transmembrane transporter (GO-Score: 0.37, GO: 0022857).

### CRISPR-Cas analysis

It has been suggested that the presence of type I-E CRISPR/cas systems can hinder the acquisition of horizontally transferred plasmids from Enterobacteriaceae [46]. Given that the plasmid identified in CA17 is normally present in Enterobacteriaceae, we examined this strain alongside CA39 and CA51 for the presence of active CRISPR/cas systems. Consistent with previous observations in *Cutibacterium acnes *, the CA17 type II strain, and also strains ATCC11828 and HL110PA4, encoded type I-E CRISPR/cas, while strains CA39, CA51 and other type I control strains appeared to lack evidence of a complete CRISPR/cas system.

## Discussion

Our analyses confirmed that CA39 and CA51 were *Cutibacterium acnes* subsp. *acnes* type IA_1_ strains with the MLST_8_ genotype ST1, as originally described by Zhang *et al*. [21]. However, we found that strain CA17 was previously misidentified [21] and is a *Cutibacterium acnes* subsp. *defendens* strain with MLST_8_ genotype ST153; this type II subsp. classification was corroborated by SLST analysis and whole genome phylogeny. The association of strains from the type IA_1_ phylogroup of *Cutibacterium acnes* with acne is well described [7, 47–49], but type II strains are generally not considered to play a role in the pathophysiology of the condition despite their presence on acneic skin [48]. Indeed, metagenomic studies have shown RT6 type II strains to be strongly associated with skin health, although CA17 belongs to the RT2 genotype [49]. Furthermore, the majority of antibiotic resistant strains isolated from acne patients cluster within the *Crep. acnes* subsp. *acnes* (type I) grouping, although antibiotic-resistant type II strains have been described in some instances [50, 51].

While all three CA strains displayed moxifloxacin resistance, the underlying mechanisms driving this phenotype have not been identified [21]; moxifloxacin resistance was based on an MIC of 2 mg l^−1^ [21] which was also previously used in other studies as a resistance breakpoint for this antibiotic in *Cutibacterium acnes* [52]. Resistance to fluoroquinolones is mostly due to alterations in the target DNA gyrase and topoisomerase IV genes, and amino acid substitutions in *gyrA* are also the major mechanism responsible for resistance in *Cutibacterium acnes* [19, 20, 53]. Indeed, on the basis of RGI analysis we identified a previously described S101L ‘deleterious’ mutation within the QPDR region of the CA37 and CA59 *gryA* gene which has been associated with high levels of quinolone resistance in other *Cutibacterium acnes* [20]. This mutation therefore helps to explain the resistance pattern seen in these strains to moxifloxacin. With CA17, we also observed a number of different *gyrA* missense mutations (A670E, E761R and Q858K) relative to our control strains covering different phylotypes of the bacterium, but only one (E761R) was classified as ‘deleterious’ on PROVEAN analysis. Although this mutation was outside the QPDR region, our comprehensive mapping of DNA gyrase and topoisomerase IV genes (*gyrA*, *gyrB*, *parC* and *parE*), as well as the entire genome sequence, could not identify any other rationale for the observed resistance. The prevalence of quinolone resistant-causing SNPs outside the QRDR region is unclear as these locations are understudied in this context, but such mutations have been previously reported in *Mycobacterium tuberculosis* [54, 55]. In the future, it will be interesting to observe if the *gyrA* allelic variants associated with resistance, and particular the E761R SNV, are found in other *Cutibacterium acnes* strains or if it appears a sporadic mutation confined to CA17.

Although all CA strains have wild-type 23S rRNA sequences, CA37 and CA59 were previously found to contain the *erm(X*) gene which likely explained their MLSB resistance profile [21]. As no clear explanation for MLSB resistance in strain CA17 was previously found, its genetic background was extensively investigated for any genetic determinants that could explain this phenotype. On the basis of our analyses, the only potential candidate that could be identified was a multidrug efflux YfmO protein similar (43 % sequence identity) to the known multidrug efflux YfmO transporter originally identified in *Bacillus subtilis* [56]. In *B. subtilis* the YfmO multidrug efflux protein is part of the YfmOP operon. The Helix-Turn-Helix (HTH) transcriptional regulator YfmP, part of the MerR family, controls the expression of the multidrug export transporter YfmO which functions as an exporter of toxic metal ions (cadmium, copper) as well as antibiotics. Structural and functional homologues of the YfmO transporters have proven to confer high levels of resistance to MLSB antibiotics in *Listeria monocytogenes* (36.6 % amino acid identity) (MdrL gene; multidrug efflux transporter of *Listeria *) [57] and *Mycobacterium smegmatis* (hypSO operon for HOCl; rifampicin and erythromycin efflux) [58]. Upon further inspection, the *yfmO* gene was found to be present in all *Cutibacterium acnes* strains, but the protein sequence in CA17 was unique in amino acid composition with the exception of the type II strain HMSC069G10 for which no phenotypic data is currently available. Compared to our antibiotic-sensitive type II control strains, we found a number of mutations of which A170T was predicted to have a strong effect on the protein’s structure. Virtually nothing is known about efflux pumps in *Cutibacterium acnes *, but as the *yfmO* efflux gene is present in all strains, a large proportion of which are likely sensitive to MLSB, we can hypothesise that the protein has low or no interactions with MLSB antibiotics in the majority of cases. However, due to the A170T SNP in CA17 there may be an enhanced affinity for MLSB drugs in this instance, thus explaining the observed phenotype. Furthermore, by closely inspecting the CA17 genome we identified a MerR family transcription repressor (PIS94143.1) which shows 39 % amino acid identity with the HypS repressor of *Mycobacterium smegmatis *. The above observations could suggest the presence of a yet uncharacterised redox sensitive operon, similar to the one in *Mycobacterium smegmatis *.

The most striking observation from our studies was the identification of a small, circular (4715 Kb) non-conjugative, colRNAI type plasmid (delegated pCA17) in strain CA17. This plasmid, which formed a complete contig, was almost identical to the previously described 4.8 kb plasmid pYU39 from the MDR resistant, human invasive, *Salmonella enterica* strain YU39, as well as other similar plasmids conserved across Enterobacteriaceae. The transfer of plasmids between Gram-positive and Gram-negative bacteria is rare, but it has been described before although not in the context of Cutibacteria [59]. The rarity of such events does raise the question of whether pCA17 is in fact a sequence contaminant. The presence of plasmids in molecular biology and next generation sequencing reagents has been described and there are many points in the laboratory workflow where contamination could occur leading to incorrect conclusions about an organism’s biology [60, 61]. Furthermore, strain CA17 represents a type II or *Cutibacterium acnes* subsp. *defendens* strain which, in contrast to *Cutibacterium acnes* subsp. *acnes* (type I) and *Cutibacterium acnes* subsp. *elongatum* (type III) organisms, contains an active CRISPR/Cas system which ‘defends’ against the acquisition of foreign DNA from viruses and plasmids [49]; this is actually the basis of the subsp. name ‘*defendens*’. In accordance with this, CA17 was found to contain a complete subtype I-E CRISPR/Cas system based on the classification of Makarova *et al*. [62]. Despite this, we could not find any evidence of sequences from this plasmid in the other CA strains, which we presume were sequenced at the same time, thus weakening the contamination hypothesis, and plasmids with signatures of conjugative transfer have previously been found in type II strains despite being CRISPR/cas-positive [63]. How CA17 could have obtained the plasmid by HGT is unclear, but the lack of a conjugation system would indicate transformation or possibly transduction. While a dominant member of the microbiota on the skin, *Cutibacterium acnes* can be present in the gastrointestinal tract so there may be potential opportunity for *Cutibacterium acnes* to acquire pCA17 in such an environment if its presence is indeed naturally acquired. There is no evidence that pCA17 plays any role in the MDR profile of CA17, although it could potentially contribute to enhanced virulence or other cellular process that enhance survival. Our annotation identified five protein-encoding regions and one non-coding RNA gene. While most were related to mobilisation or regulatory proteins, one open reading frame encoded a hypothetical protein. On the basis of some preliminary analysis, this protein appeared to be an uncharacterised transmembrane transporter containing cytoplasmic, transmembrane helix, extra-cellular and signal peptide fragments. Motif analysis also identified a EF-hand Ca^2+^-binding domain which may indicate that the protein has a role in homeostatic processes [64].

Although algorithms for mining of microbial genomes have greatly improved over the years, there are still limitations with such *in silico* analyses. These include the small fraction of genes that are functionally validated, as well as difficulties in verifying the function of mobile genetic elements without laboratory-based studies. Furthermore, in relation to *Cutibacteria*, there is a profound lack of organism-specific bioinformatics tools that would aid with the identification of functionally validated genomic determinants of virulence or resistance.

In summary, this study has characterised genetic mechanisms underlying the MDR of three *Cutibacterium acnes* strains previously isolated from patients with acne [21]. We identified previously described and novel *gyrA* amino acid substitutions that could explain fluoroquinolone resistance, and a putative efflux pump that could potentially play a role in MLSB resistance under certain circumstances. Most notably, we identified a non-conjugative Enterobacteriaceae originating plasmid borne contig in one of the strains that could, if confirmed, represent a striking and rare case of HGT between Gram-negative and Gram-positive organisms. The presence of this plasmid, if natural, many contribute to cellular processes that enhance survival or virulence. These *in silico* observations will now provide a platform for laboratory studies with these strains to validate our whole genome results.

## Supplementary Data

Supplementary material 1
