# Supplementary material for: Genetic determinants of antimicrobial resistance in three multi-drug resistant strains of Cutibacterium acnes isolated from patients with acne: a predictive in silico study
Source: Access Microbiol. 2022 Aug 11;4(8):acmi000404. doi: 10.1099/acmi.0.000404 (PMC9484663; doi:10.1099/acmi.0.000404)
Supplement: Supplementary material 1 [file acmi-4-404-s001.pdf]

Figure S1. Molecular evolution cladogram of concatenated gene sequences (4,253) from all STs currently presented in the *C.acnes* MLST database. Sequence input order was randomised and bootstrapping resampling statisctis were performed using 500 datasets. ST1 and ST153 are indicated alongside ST4, ST5, ST7, ST22, ST27 and ST70 from our reference strains. Bootstraps ≥70% (circles). Different colours represent the major phylogroups from which our strains were selected for analysis.

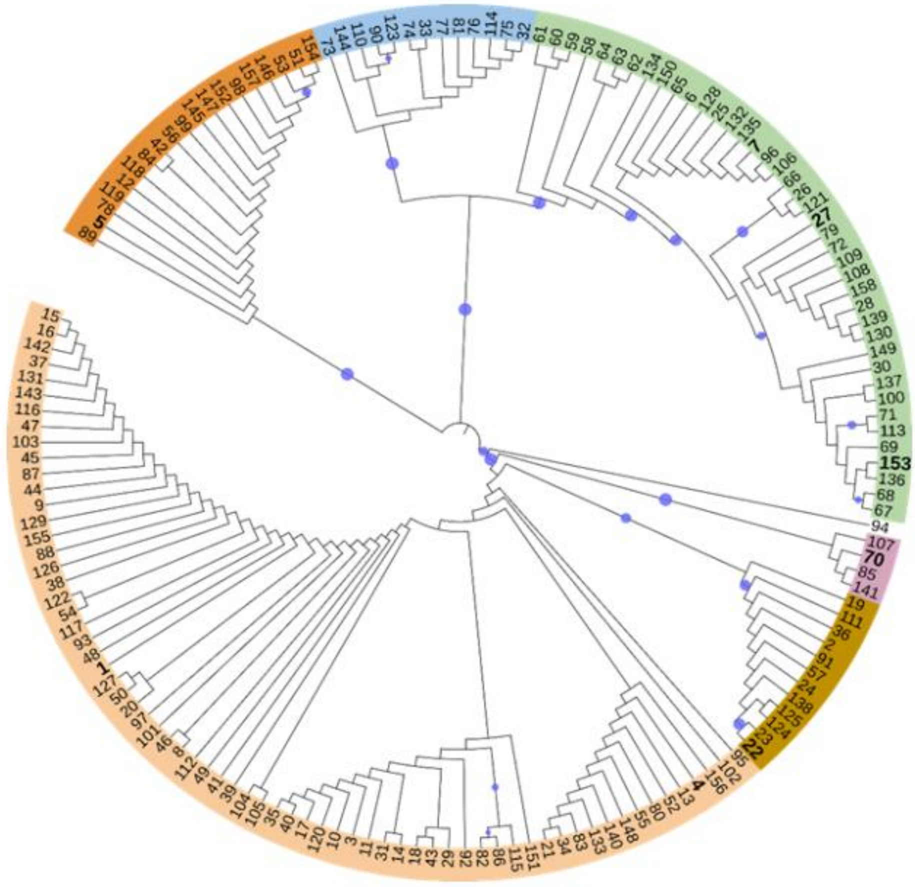

Table S1. The key strains, proteins and plasmids referred to in this study.

Strain ID	Biosample ID
CA17	SAMN07259472
CA39	SAMN07268976
CA51	SAMN07268412
HL025PA1	SAMN00189202
P.acn17	SAMN02602999
KPA171202	SAMN08348521
PRP38	SAMN02469319
ATCC11828	SAMN02602997
HL110PA3	SAMN00189249
HL110PA4	SAMN00189250
**Protein**	**GeneBank accession number**
YfmO (CA 17)	PIS93044.1
YfmO(*B.subtilis*)	O06473.1
MerR family transcriptional repressor (CA 17)	PIS94143.1
HypS repressor (*M.smegmatis*)	ABK72338.1
pCA17 hypothetical protein	PIS94559.1
**Plasmid**	**GeneBank accession number**
pCA17	NJFY01000001.1
pYU39	CP011433.1
